# Exploring postmortem practices for cardiac device interrogation in the UK

**DOI:** 10.1136/heartjnl-2025-326759

**Published:** 2025-11-12

**Authors:** Holly Morgan, Tesfamariam Betemariam, Matthew Ryan, Rachel Bastiaenen, Pier D Lambiase, Aldo Rinaldi, Divaka Perera

**Affiliations:** 1 Cardiovascular medicine, King’s College London, London, England, UK; 2 Guy's and St Thomas’ Hospitals NHS Trust, London, England, UK; 3 University College London, London, England, UK

## Abstract

**Introduction:**

Ascertaining the cause of sudden death is often difficult, even in patients with a history of heart disease. Device interrogation is routinely carried out to determine the nature of symptoms or aborted sudden death episodes in patients with cardiac implantable electronic devices (CIED), but it is not clear how often this is done postmortem, and if so, how this information is recorded and used by clinical teams and coroners.

**Methods:**

We determined the proportion of deaths with a CIED in situ, the capacity for and frequency of postmortem device interrogation and when done, how the information was used over a 5-year period in the UK by surveying 173 National Health Service (NHS) trusts via a freedom of information request.

**Results:**

A response was received from 83 (48%) NHS sites, 75 (90%) of which reported having both a mortuary and cardiac physiology department onsite. During the period 2019–2024, each mortuary handled 2400±1094 deaths per annum, of which an estimated 5±2% had a CIED in place. Of those with cardiac physiology on site, only 15 (20%) reported routine postmortem device checks were performed. If such a check was conducted, 3 out of 15 (20%) responded that findings were documented in the medical records and 2 out of 15 (13%) stated information was relayed to the medical team.

**Conclusion:**

Although 1 in 20 patients who present to NHS mortuaries have a CIED in situ, routine postmortem checks are performed rarely and inconsistently documented. Prospective studies are warranted to determine the feasibility and utility of standardised postmortem device interrogation.

## Introduction

 Accurate identification of a patient’s cause of death can frequently pose a challenge, even in individuals with established cardiovascular disease. A significant proportion of these patients have a cardiac implantable electronic device (CIED) in situ, including pacemakers and cardioverter-defibrillators (ICDs). An estimated 50 000 CIEDs are implanted each year across the UK.^1 2^ Prior studies have shown that postmortem interrogation of CIEDs provides invaluable insights into cause of death, particularly in the setting of sudden unexpected death (SUD). As well as identification of arrhythmias, device malfunction and therapy failure can also be detected, which are crucial for improving patient safety.^2^,^6^

Despite this, routine postmortem checks are not standard practice in many countries. Even in the setting of randomised controlled trials, where accurate recording of cause of death is vital for comparing outcomes, such checks frequently do not occur.^7^ This absence results in a loss of potentially vital information as well as missed opportunities to guide family counselling and screening.

We aimed to assess the frequency of routine postmortem device checks by investigating variations in practice across National Health Service (NHS) trusts in the UK, as well as exploring the feasibility of the introduction of mandatory postmortem CIED checks for SUD.

## Methods

Freedom of information requests were issued in February 2024 to 173 NHS trusts, containing an 8-question survey (online supplemental table S1). The survey sought information on hospital mortuary volume, presence of a cardiac physiology department, prevalence of routine postmortem device checks and the practice of documenting and communicating device data to clinical teams (online supplemental table S1). A CIED was defined as any cardiac device, including pacemaker, ICD or implantable loop recorder. Patients and the public were not involved in the design, conduct or reporting of this study.

Categorical data are presented as counts (and percentages) and continuous data as mean±SD. The unpaired t-test and χ^2^ test were used to study between-group differences. Qualitative thematic analysis was undertaken on open box responses. Formal ethical approval was not required as the project was considered to be a service evaluation.

## Results

**Figure 1:**
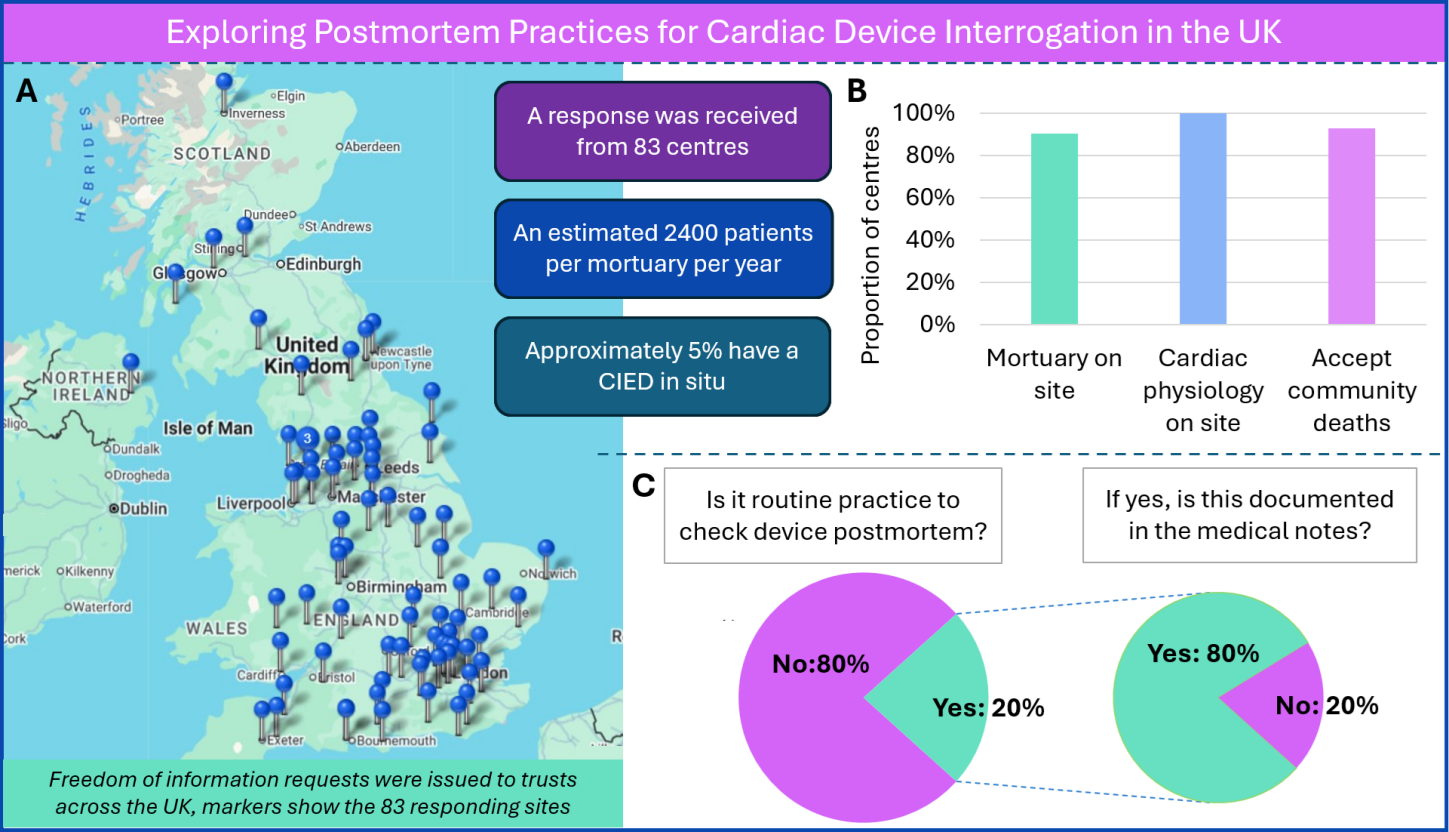
Responding trusts across the UK and key responses. CIED, cardiac implantable electronic device.

At 90 days post-request, 83 sites (48%) had responded to the freedom of information request (figure 1A). Eight sites reported not having an on-site mortuary and were therefore excluded from the analysis. Within the 75 sites with a mortuary on site, 70 (93%) reported accepting patients from the inpatient setting as well as the community and 100% had a cardiac physiology department (figure 1B).

Mortuaries reported handling 2400±1094 deaths per year, approximately 5% (0.5–20%) of which involved patients with a CIED. Routine postmortem cardiac device checks were performed in 15 (20%) hospitals. There was no association between reporting that routine checks were undertaken and the type (yes 27% vs no 35% tertiary, p=0.54) or size of hospital (yes 706 beds vs no 616 beds, p=0.34).

Out of these 15, 3 (20%) respondents stated findings were documented in the medical records and 2 (13.3%) respondents stated these were communicated to the medical team (figure 1C).

Open-text responses from centres who did not undertake checks routinely indicated that postmortem device interrogation occurs only in specific circumstances, such as under the direction of the clinical team, coroner or pathologist. Responses stated this was most likely to occur in the setting of a sudden or unexpected death, or during a postmortem. Five responses stated that interrogation is performed at the time of ICD deactivation. While most reported findings would be documented and/or fed back to clinical or legal teams, one respondee noted that findings would not routinely be entered into medical records unless a check had been specifically requested (online supplemental table S2).

## Discussion

Our study highlights a significant paucity of postmortem examination of CIEDs. The low rate of routine postmortem checks contrasts sharply with the high prevalence of cardiac physiology departments. It could be that the absence of an established protocol and mandate is the primary obstacle.

If a patient has a suspected cardiac arrhythmia and has a CIED in situ, the first step in management is to undertake a device interrogation to elucidate if an arrhythmia has occurred. However, when an event is terminal, the presence of the device appears to be forgotten.

A key challenge in clinical practice is establishing the definite cause of death in patients who have SUD. Distinction between arrhythmic and non-arrhythmic cause is essential, not only for clarity for national reporting and families, but also for clinical trials which include arrhythmic outcomes, where misclassification could skew results and lead to incorrect conclusions.^8 9^ A recent meta-analysis highlighted the value of postmortem CIED interrogation as a useful adjunct to autopsy, particularly in cases of SUD.^10^

Several studies have also identified the importance of postmortem CIED interrogation in uncovering device-related issues that may not have been previously apparent.^8^ One meta-analysis of 3194 deceased CIED carriers found potentially fatal device malfunctions in 12% of cases.^3^ This practice also aligns with broader efforts to improve patient safety and device reliability, including detection of suboptimal therapy delivery.^8 11^

A key aspect of SUD is in advising and screening first-degree relatives who may also be at risk. The inherited cardiac conditions community are currently running a pilot programme across the UK, supported by NHS England and the British Heart Foundation, aimed at improving communication between the coronial services and clinical services, as well as establishing consistent postmortem and pathology practices for tissue retention and genetic testing.^12^ A standardised protocol for postmortem CIED checks should be incorporated into this process and ensure collaboration between national health bodies, healthcare providers, regulatory bodies and professionals.^3^

Although a high proportion of centres reported having both an onsite mortuary and cardiac physiology, the extent to which this infrastructure could support routine practice remains uncertain. It may be that the primary barriers are not related to limitations in facilities or expertise, but rather a lack of standardised procedures. However, it is essential to consider the potential impact on cardiac physiology department workload. Currently, physiologists only routinely attend postmortem for ICD devices, to disable tachyarrhythmia therapies.^13^ Expanding postmortem interrogation to include all CIEDs would represent an increase in workload for understaffed departments already managing high clinical demands.^14^ Any future implementation of a routine postmortem interrogation protocol would need to carefully consider these resource implications.

Although this analysis did not include public mortuaries, which make up approximately 10% of UK mortuaries and accept deaths that occur in the community, to our knowledge these centres transfer patients with implantable defibrillators to a hospital mortuary for deactivation. There is therefore a pathway in place should a death be sudden and a CIED be present. Notably approximately 50% of deaths occur outside of hospital settings.

The limitations of our study include its retrospective questionnaire design and therefore responses may be estimates. Moreover, the study reflects a best-case scenario in terms of infrastructure, as it was conducted in settings where mortuary and cardiac device services were co-located and specialist expertise readily available. This may not be representative of worldwide practice. We also acknowledge that prospective studies are needed to determine how often device interrogation data leads to a revised cause of death and whether such information influences subsequent clinical decisions.

## Conclusion

This study indicates that although a notable cohort of patients with CIEDs pass through hospital mortuaries every year, most centres do not have a standardised procedure for postmortem device interrogation. To bridge this gap in practice, it is imperative that regulatory bodies and national professional societies collaborate to develop and implement a national standard for postmortem CIED interrogation. Prospective studies are warranted to determine the feasibility of implementing routine postmortem CIED interrogation in varied clinical settings, and to assess impact on diagnostic accuracy and subsequent clinical decision-making.

## Supplementary material

10.1136/heartjnl-2025-326759 online supplemental file 1

## Data Availability

Data are available upon reasonable request.

## References

[1] (2021). National audit of cardiac rhythm management (NACRM): national cardiac audit programme. https://www.nicor.org.uk/national-cardiac-audit-programme/previous-reports/cardiac-rhythm-management-1.

[2] Raatikainen MJP, Arnar DO, Merkely B (2017). A Decade of Information on the Use of Cardiac Implantable Electronic Devices and Interventional Electrophysiological Procedures in the European Society of Cardiology Countries: 2017 Report from the European Heart Rhythm Association. EP Europace.

[3] Paratz ED, Block TJ, Stub DA (2022). Postmortem Interrogation of Cardiac Implantable Electronic Devices: A 15-Year Experience. JACC Clin Electrophysiol.

[4] Kirkpatrick JN, Ghani SN, Burke MC (2007). Postmortem interrogation and retrieval of implantable pacemakers and defibrillators: a survey of morticians and patients. J Cardiovasc Electrophysiol.

[5] Nikolaidou T, Johnson MJ, Ghosh JM (2018). Postmortem ICD interrogation in mode of death classification. J Cardiovasc Electrophysiol.

[6] Lacour P, Buschmann C, Storm C (2018). Cardiac Implantable Electronic Device Interrogation at Forensic Autopsy: An Underestimated Resource?. Circulation.

[7] Lackermair K, Fischer F, Manhart J (2022). Determination of time of death by blinded post-mortem interrogation of cardiac implantable electrical devices. Sci Rep.

[8] Li H, Axtell K, Biehl M (1996). Sudden death in patients with implantable cardioverter-defibrillators. Am Heart J.

[9] Pires LA, Lehmann MH, Steinman RT (1999). Sudden death in implantable cardioverter-defibrillator recipients: clinical context, arrhythmic events and device responses. J Am Coll Cardiol.

[10] Fede MS, Compagnucci P, Montana A (2024). Forensic perspectives on postmortem CIED interrogation: A systematic review and meta-analysis. Forensic Sci Int.

[11] Tseng ZH, Hayward RM, Clark NM (2015). Sudden Death in Patients With Cardiac Implantable Electronic Devices. JAMA Intern Med.

[12] British Heart Foundation (2023). NHS and coronial service sudden unexpected death programme evaluation interim report nhs-coronial service sudden cardiac death programme-interim evaluation report. https://www.bhf.org.uk/-/media/files/for-professionals/healthcare-professionals/nhs-c-sud-interim-evaluation-report-final.

[13] Medicines and Healthcare products Regulatory Agency (2014). Implantable cardioverter defibrillators (icds) - disable all high voltage shock therapies before you remove icd. https://www.gov.uk/drug-device-alerts/medical-device-alert-implantable-cardioverter-defibrillators-icds-disable-all-high-voltage-shock-therapies-before-you-remove-icd.

[14] (2016). Cardiac physiology workforce options analysis: executive summary. https://bhrs.com/wp-content/uploads/2019/03/180418-sp-Cardiac-Physiologist-Workforce-Options-Analysis-May-2016.

