# Supplementary material for: Exploring postmortem practices for cardiac device interrogation in the UK
Source: Heart. 2025 Nov 12;112(14):e326759. doi: 10.1136/heartjnl-2025-326759 (PMC13311938; doi:10.1136/heartjnl-2025-326759)
Supplement: online supplemental file 1 [file heartjnl-112-14-s001.pdf]

Supplementary data

Table S1: Freedom of information questionnaire

Choose an item drop down: Yes, No

Q1	How many patients pass through your morgue each year?	
Q2	Approximately what proportion of these have a cardiac implantable device in situ? (PPM, ICD, ILR)	
Q3	Does the hospital morgue also take deaths from the community, or is it for inpatients only?	
Q4	Is there a cardiac physiology department on site at your hospital?	*Choose an item.*
Q5	If a patient has a cardiac device in situ, is it routine practice for a device check to be undertaken after death?	*Choose an item.*
Q6a	If yes, is the information regarding rhythm/therapies at the time of death routinely added to the patient's notes/hospital record?	*Choose an item.*
Q6b	If yes, is the information regarding rhythm/therapies at the time of death routinely passed on to the clinical team?	*Choose an item.*
Q7	If no and this is not routine practice, are there ever exceptions to this, i.e., occasions where a post-death device check is requested by the clinical team?	*Choose an item.*
Q8	If yes, please elaborate (for example, how often or under what circumstances this occurs).	

Table S2: Thematic analysis of 56 open box responses

**Rarity of Post-Mortem Device Checks**
• Multiple responses highlight that checks are very infrequent – "happens very occasionally - less than once a year," "I have not been asked since 2020," "extremely rare," "once in four years"<br>• In the majority of centres device interrogation after death is not routine practice; it occurs only in exceptional circumstances.
**Situations When Checks Are Performed**
**1. Unexplained or Sudden Death**<br>• Checks may be requested if the cause of death is uncertain, "sudden," or "unexplained"<br>• Check may occur if arrhythmia is the suspected cause of death, "if there is any concern over device function or rhythm," "would not occur if death was expected for example cancer"
**2. Coroner/Post-Mortem Involvement**<br>• Interrogation is commonly carried out when specifically requested by the coroner<br>• One trust mentioned these are performed in context of hospital post-mortems, but not routinely for "fiscal cases"
**3. Device Deactivation for Safety**<br>• ICDs are always deactivated after death for safety, especially prior to cremation or removal by mortuary staff. Device interrogation occur during this process.<br>• Confirmation of deactivation is typically documented and communicated to the mortuary.
**4. Specific Requests**<br>• Checks may occur upon request from coroners, clinicians, police or family members.<br>• Funeral directors ask for confirmation of device presence/deactivation
**Types of Devices Checked**
• **Implantable Cardioverter Defibrillators / CRT-D:**<br>○ Five sites reported these are routinely interrogated on deactivation, "The exception is ICDs – the HV part of the device is programmed OFF and we would collect any events that may have been recorded"<br>○ If any arrhythmia detected this is then fed back to clinical teams
• **Pacemakers / Implantable Loop Recorders:**<br>○ Many responses stated these are not routinely checked post-death<br>○ Sometimes interrogated in unexplained/sudden death or at clinical request<br>○ Responses included, "This has occurred once in the last five years"<br>○ One response stated they would interrogate if death occurred soon after implant
**Documentation and Reporting**
• Device checks generate reports which are:<br>○ Passed on to the mortuary team<br>○ Added to the patient's hospital record and shared with clinical teams<br>○ Not always formally documented; "this would only occur if the check had been requested by the medical team", "information on deceased patients cannot be added to the electronic record"
**Staff Roles and Processes**
• Cardiac physiologists or cardiology teams perform interrogation and deactivation<br>• Mortuary/pathology staff identify and remove devices but generally do not interrogate them; one response stated their mortuary staff were trained in deactivation.<br>• Funeral directors request confirmation of device removal
